# A population-based study on infertility and its influencing factors in four selected provinces in Iran (2008-2010)

**Published:** 2014-08

**Authors:** Marzieh Rostami Dovom, Fahimeh Ramezani Tehrani, Mehrandokht Abedini, Golshan Amirshekari, Somayeh Hashemi, Mahsa Noroozzadeh

**Affiliations:** 1 *Reproductive Endocrinology Research Center, Research Institute for Endocrine Sciences, Shahid Beheshti University of Medical Sciences, Tehran, Iran.*; 2 *Department of Family Health, Ministry of Health and Medical Education, Tehran, Iran.*; 3 *Endocrine Research Center, Research Institute for Endocrine Sciences, Shahid Beheshti University of Medical Sciences, Tehran, Iran.*

**Keywords:** *Infertility*, *Prevalence*, *Female*, *Sterility*, *Populationbasedstudy*

## Abstract

**Background:** Infertility has a varied impact on multiple dimensions of health and functioning of women.

**Objective:** We aimed to identify the burden of infertility and its influencing factors based on a population based study conducted in four provinces of Iran.

**Materials and Methods: **A sample of 1126 women, aged 18-45 years, was selected using the multi stage, stratified probability sampling procedure; those met the eligibility criteria were invited for further comprehensive interview. This study used the definition of infertility proposed by World Health Organization “the woman has never conceived despite cohabitation and exposure to pregnancy for a period of 1 year”.

**Results** **: **The overall prevalence of lifetime infertility and current primary infertility were 21.1% (95% CI: 18.4- 23.8) and 6.4% (95% CI: 4.8-8) respectively. The probability of first pregnancy at the end of 2 years of marriage was 94% for all ever-married women. Infertility were observed as significantly higher among women age 31-35 (OR: 4.6; 95% CI: 1.9-11.5; p=0.001) and women with more than 9 years of education (OR: 2.8; 95% CI: 1.5-3.3; p<0.0001).

**Conclusion:** The necessities of modern living have compelled many women to postpone childbearing to their late reproductive years; however they must be informed of being at risk of infertility with ageing.

## Introduction

Infertility affects approximately 60-80 million couples around the world and is still increasing (1-3). A demographic study in 2002 by the World Health Organization (WHO) on developing countries (except China) indicated that 186 million women have been infertile (4). The prevalence of current infertility in developed and less developed countries, based on a systematic review, was between 3.5-16.7% and 6.9-9.3% respectively (2). Infertility is not only a health problem but also a social and emotional problem, especially in some cultures and sometimes it leads to divorce (   5 , 6). Identification of the burden of infertility in each country has a critical role in evidence based decision making, however to achieve this goals the accurate calculation of primary infertility rate is significantly important. Lack of access to a universal definition for primary infertility and precise methodology to determine infertile women and the population exposed to the risk of fertility have a great impact on the estimated infertility prevalence and its influencing factors.

Another study has reported the prevalence of primary infertility in developed and less developed countries at 6.6-26.4% and 5-25.7% respectively (7). The prevalence of primary infertility has been reported in China 9%, in America 10-15%, in Siberia about 16% and in Australia 19% (6). The rate of primary infertility in various reports has been reported 8-21.9% (8). This figure is significant because infertility in the world is estimated 8-12% by the WHO (9). Some studies in Iran have estimated current primary and lifetime infertility between 2.8-3.4% and 21.9-24.9% respectively (10-13). There is no report about secondary infertility rate in any ones.

The estimations of diseases prevalence are used to surmise the burden of disease, to estimate the need rate of healthcare services, to compare the incidence of disease in different societies and also to test the trend of disease. In such estimations, the prevalence rate of infertility as a health problem is not an exception. Infertility treatment costs impose a large economic burden on health systems as well as families and social insurance systems often rebuff infertility treatments due to their high costs (14). It seems that the difference in prevalence of infertility in different countries and even in a region in addition to different biological and epidemiological factors is rooted in definitions used in each study (2). Infertility is divided into three main groups of primary infertility, secondary infertility and lifetime infertility. The primary infertility is defined differently based on the variable of waiting period length. In clinical definition, infertility is referred to lack of fertility after one year of consistent and unprotected sexual intercourse (15). 

This term is also considered two years by epidemiologists (15, 16). They believe that many couples who are considered infertile through assuming one-year period, with a little patience, develop into fertile in the second year, therefore, they are more circumspect in defining infertility (8, 16). In adjusted definition of infertility in 2008 by the American Society for Reproductive Medicine (ASRM), infertility is referred to as the failure to become pregnant after 12 months or more of continuous and unprotected sexual intercourse and it is suggested to begin the evaluation and treatment based on clinical history and physical examinations in over 35 years women, after 6 months (17). At the same time, the demographers define infertility as failure to give live birth in women who had active sexual intercourse not using any contraceptive method (18). In addition to biological and organic factors inducing infertility, several demographic, socio-economic and anthropometric factors have been reported as the factors that mainly affected infertility. Age, age of marriage, place of living, race, adiposity has influence on infertility, in some studies (8, 19). 

We aimed to investigate the prevalence of infertility in a population-based study of 18-49 years old Iranian women recruited from urban areas of four provinces of Iran and specify the effective underlying factors.

## Materials and methods

This study was a cross-sectional study. After obtaining approval from the Ethics Committee of the Research Institute for Endocrine and Metabolism Sciences of Shahid Beheshti University of Medical Sciences, the study population was selected by using stratified, multistage probability cluster sampling method, with a probability in proportion to size procedure, was used. The subjects of the present study were recruited using cluster sampling method from 4 randomly selected provinces of Iran (Golestan in East, Qazvin in North, and Kermanshah in West and Hormozgan in South). The detailed protocol of sampling method was reported in another published article (20). Sample size was calculated based on these parameters: p=0.085 [5], a=0.95, d=0.025, cluster design effect =2 and a non-response rate =0.15.

A total number of 1126 non-menopausal women age 18-49 were recruited for the purpose of the present study after obtaining written consent. We excluded unmarried women (n=105), those who had never have willingness for pregnancy (n=43), those with less than one year time from their pregnancy willingness (n=34), widows or divorced women (n=36) and those whose marriage long were less than one year (n=20). A standard comprehensive questionnaire (The content validity of the questionnaire was assessed by 15 gynecologists and reproductive health experts of Shahid Beheshti and Tehran Medical Science universities. The reliability of the questionnaire was assessed using test-retest and inter-rater methods, both confirmed by r=0.91 and r=0.85, respectively.) including information on socio-demographic and reproductive variables was completed, during face-to-face interviews by trained staff members of local medical universities and a trained supervisor monitored the process in each district.

Current primary infertility refers to couples who have not ever become pregnant after at least 1 year of unprotected intercourse (according to the answer to these question "how long after you have attempted to get pregnant? Or, if they had used contraception “how long after stopping your contraceptive method you became pregnant for the first time? ") and "have you ever been pregnant?" (13). Secondary infertility refers to couples who have been pregnant at least once, but are not able to get pregnant when they attempt to conceive for the next time (according to the response to the question “have you had pregnancy delay for your second pregnancy (over one year passed from your tendency to become pregnant or in case of using contraceptive methods more than one year after outage of method). Lifetime infertility was defined as having any delay (more than one year) to get pregnant during their life regardless of whether or not has the child now. The question was used to determine lifetime infertility rate. 

**Statistical analysis**

The data were analyzed by Statistical Package for the Social Sciences, (SPSS) version 15.0, SPSS Inc, Chicago, Illinois, USA. The concepts of descriptive statistics (absolute frequency, relative frequency, mean, standard deviation and median) as well as calculation of 95% confidence interval were employed considering sampling design. T-tests were used to compare the means of continuous variables with normal distribution and chi^2^ statistical test was used to compare qualitative variables. 95% confidence interval was used to estimate prevalence rate of primary and secondary infertility. In all tests, the significance level of p<0.05 was considered. The multiple logistic regression tests were used to find the relationship of independent factors associated with the dual dependent variable of primary infertility. 

## Results

Among the 1126, premenopausal women age 18-45 participating in this study, 888 persons were met our eligibility criteria for the purpose of the present study. 

The demographic and anthropometric characteristics of the study participants are presented in table I. The mean±SD of age of women was 34.8±6.9 years; about 1/3 of women had diploma level of education and 8.2% of women were illiterate. Overweight, defined as body mass index of 25-29.9 kg/m^2^, was observed in 41.7% of women. Overall, among 888 women had attempt for more than one year for pregnancy, 93.5% were ever pregnant and 6.4% ones never get pregnant (current primary infertility). Among women with delay in first pregnancy 103 (56%) have been seeking treatment. The prevalence of lifetime infertility, current primary infertility and secondary infertility in this study was 21.1% (95% CI: 18.4-23.8), 6.4% (95% CI: 4.8-8) and 7.8% (95% CI: 6-9.6) respectively. cause of primary infertility, after unknown causes (39.4%), were related respectively to an-ovulation (25.7%), male factors (17.3%), uterine causes (8.7%) and tubal factor (2.9%). The main causes of secondary infertility were unknown causes (30.4%), an-ovulation (29.3%), male factors (26.2%), tubal factor (7.7%) and uterine problems (7.2%). 

There were significant statistical difference between average age of marriage for women with a history of primary infertility and women with normal fertility (34.3±7.1 vs. 36.4±6.2, respectively (p=0.001), however, no significant difference was found between the average ages of marriage of their partners. There was a significant statistical difference between average years of schooling for women with a history of primary infertility and average years of schooling for women with normal fertility (8.6±4.4 vs. 7.01±4.2 years, p<0.001) (Table II). Using the logistic regression model, the odds ratio (95% CI) of infertility for women age 31-35 and those age >35 compare to women age <25 were 4.6 (1.9-11.5) and 2.9 (1.2-7.1), respectively. 

Among all other variables inserted to model, besides women’s age, the infertility was more observed among those with more than nine years of education (OR: 2.8; 95% CI: 1.5-3.3), (Table III). There was no significant statistical difference between infertility in reason of male factor as a sub categorical cause of infertility and spouse’s age.

**Table I:** The Characteristics of study participants

**Characteristics **		**Number**	**%**
Age( year)			
	18-30	257	28.9
	21-40	418	47.1
	≥41	213	24
Menarcheal age			
	Mean (SD)	13.4 (1.6)	-
	Median	13	-
Year of education			
	0 (illiterate)	72	8.3
	1-9	429	48.9
	10-12	288	32.8
	>12	88	10
BMI (kg/m^2^)			
	<24.99 (normal)	255	30
	25-29.99 (over weight)	355	47.1
	>30 (obese)	241	28.3
Spouses marriage age Means(SD)		24.7 (5.9)	----

**Table II:** The Comparison of characteristics of infertile women with fertile women

	**Primary infertility**		**p-value**
	**Infertile women**	**Fertile women**	
Age at interview(year)	34.3 ± 7.1	36.4 ±6.2	0.001
Age at current marriage(year)	20.1 ± 3.4	19.3 ± 3	0.001
Spouse’s age at marriage time(year)	24.8 ± 5.3	24.4 ± 6.5	0.33
Education years	8.6 ± 4.4	7.01 ± 4.2	0.000
Body Mass Index(kg/m2)	28.4 ± 4.4	27.9 ± 5.1	0.063

* Data are presented as mean±SD.

**Table III:** Odds Ratio with 95% confidence intervals for infertility in reproductive age women according to their demographic characteristics

**Characteristics **		**p-value**	**Odds Ratio (95% CI** * **)**
Age(year)			
	≤24	-	(Ref) 1
	25-30	0.136	2.1 (0.8-5.23)
	31-35	0.001	4.6 (1.9-11.5)
	≥36	0.01	2.9 (1.2-7.1)
Education (year)			
	≤9	-	(Ref) 1
	>9	0.000	2.8 (1.5-3.3)

* CI: confidence interval

## Discussion

Despite the heavy burden and impact of the infertility, estimations regarding its prevalence are limited. The reported prevalence of infertility ranges between 3.5% to 22% in various countries, depending on the recruitment process of the study population, the criteria used for its definition and the method used to estimate fertility situation of each woman (21-24). Defining the population at risk of infertility is difficult and without extra details, it is not possible for responder or even interviewers to find the true answer. The present population based study demonstrates a high prevalence of lifetime infertility among reproductive age Iranian women. Whereas the current primary infertility rate was 6.4%, however about one fifth of women (21.1%) had more than one year delay for pregnancy that forced them seeking medical treatment. 

Among various demographic characteristics, infertility has mainly affected by ageing; the problem that will be observed in modern societies which compelled many women to postpone childbearing to their late reproductive years. Our lifetime estimation of infertility is in agreement with study conducted by Barooti *et al* among women living in Tehran in reproductive age and Vahidi *et al*, however is much more than that was reported by Safari Nejad *et al* (21.1% vs. 8%)   (8, 10, 11). Recruitment strategy and various definitions for primary infertility could possibly explain part of this controversy. Infertility in other parts of theworld has different prevalence. Using the same definition, as we used, primary infertility was 15.7% in Canada compare to 3.9% in Pakistan and 9.1% in Nepal (21, 22). This difference is most considered where the probability of primary infertility in couples of industrialized countries is estimated at 10-15% (21). 

In addition to various socio-economic situation and different ethnicity, lack of uniformity for the method of calculating primary infertility rate hamper comparative studies between populations. Gurunath *et al* emphasizes that the difference in definitions used on primary infertility, even in a single population, resulted a wide range of estimation for infertility from 6.8-38.6% (16, 22, 25). Various questions have been proposed to be asked for precise estimation of infertility; among them Larsen suggested the question "How long after sexual intercourse you became pregnant?" The other questions that have been highly used for self-reported infertility are: Have had you ever experienced difficulty in conceiving”? and “How long have you unprotected intercourse for at least 2 years” (22).

The estimated prevalence of secondary infertility in the present study was 7.8% (95% CI: 6-9.6). There were also various estimations for secondary infertility in different countries from 7% in Scotland to 23% in some African countries such as Central African Republic (26, 27). This difference in the rate of secondary infertility could be partly explained by the possible situations resulted it. In some countries, it is mainly caused by genital infections; sexually transmitted infections (STI) are responsible for more than half of their secondary infertility (28). In addition to the sexually transmitted diseases another cause of secondary infertilities is due to mismanagement of previous pregnancies that will encounter women with problems in future pregnancies including insecure abortions, long-term rupture of amniotic sac, post- delivery infections as well as retention of placenta in uterus and its subsequent infections (29). 

However, unlike other studies, in this study the most common cause of secondary infertility after unknown factors, was an-ovulation (28.9%); it could be explained by the high prevalence of polycystic ovarian syndrome; 14.6% using Rotterdam definition and 8.5% using NIH criteria (30, 31). The high incidence of unexplained infertility in this study could be due to lack of specific laboratory tests to diagnose the exact cause of infertility and just relying on the statements of samples. The results of this study indicate that the age of marriage of women has a clear effect on the prevalence increase of infertility. The impact of this factor on infertility is along with physiological changes that occur in ovaries with ageing. The adverse effect of ageing on infertility has been reported in several studies, furthermore the failure rate of advanced reproductive treatments was also increased with ageing   (32-34).

There is controversy on adverse effect of ageing of men on their reproductive capacities; however it has been shown that aging in men is associated with an increase in sulfur, copper and calcium intake by sperm cells that will reduce the quality of semen and induce genomic abnormalities (13, 35, 36). However there was not any association between spouse’s age and infertility in the present study. Our study has not had enough power to investigate this issue among a sub sample of participants with male infertility. 

We found that infertility was increased about two times among women with more than 9 years of education; this adverse effect of higher education was also reported by another study (37). This adverse effect of higher education may not directly related to education but possibly it presents its effect on increasing the age of marriage or age of attempting for pregnancy; the problem that many women in modern societies are faced with. It seems that providing facilities for women those who have both motherhood and employment roles could effectively prevent this adverse effect of education and seeking career.

The main strength of the present study is its methodology, as it is a community based prevalence study carried out on an ethnically homogenous population and had an appropriate response rate of 91%. The majority of previous studies on infertility have relied upon a convenience sample of applicants (38, 39). Our study does have some limitations; we did not use medical records to identify the causes of infertility and rely on the statements of participants. Our estimation may be affected by recall bias; however fertility is an important milestone in women’s life that is well remembered by them.

## Conclusion

Infertility is a prevalent problem in our society especially among those postpone childbearing to their late reproductive years, possibly for a proper career. Providing facilities that enable women to have motherhood role along with careers could possibly diverse this alarming increasing trend of infertility.
